# Draft Genome Sequence of *Lactococcus* sp. Strain Rs-Y01, Isolated from the Gut of the Lower Termite *Reticulitermes speratus*

**DOI:** 10.1128/genomeA.00999-17

**Published:** 2017-09-21

**Authors:** Masahiro Yuki, Mitsuo Sakamoto, Hirokazu Kuwahara, Yuichi Hongoh, Moriya Ohkuma

**Affiliations:** a Biomass Research Platform Team, Biomass Engineering Program Cooperation Division, RIKEN Center for Sustainable Resource Science, Tsukuba, Japan; b Japan Collection of Microorganisms, RIKEN BioResource Center, Tsukuba, Japan; c PRIME, Japan Agency for Medical Research and Development (AMED), Tsukuba, Ibaraki, Japan; d Department of Life Science and Technology, Tokyo Institute of Technology, Tokyo, Japan

## Abstract

Here, we report the draft genome sequence of *Lactococcus* sp. strain Rs-Y01, which was isolated from the gut of a wood-feeding termite. The genome information will facilitate the study of the symbiotic functions of this strain in the termite gut.

## GENOME ANNOUNCEMENT

The complex microbiota in the gut of termites plays key roles in the digestion of lignocellulose and supply of nutrients (1–3). A previous study on the community structure of bacteria with clone library analysis of PCR-amplified 16S rRNA genes revealed the presence of bacteria in the genus *Lactococcus* represented by two previously uncharacterized phylotypes, Rs-C68 and Rs-B70, in the gut of the wood-feeding lower termite *Reticulitermes speratus* (4). We isolated a bacterial strain belonging to the genus *Lactococcus*, designated strain Rs-Y01, from the gut content of *R. speratus*. The 16S rRNA gene sequence analysis indicated that strain Rs-Y01 was closely related to Rs-B70, showing 99.9% nucleotide identity.

The genome sequencing of *Lactococcus* sp. strain Rs-Y01 was performed on the MiSeq platform (Illumina) using a MiSeq version 3 reagent kit (600 cycles). The generated reads were assembled using SPAdes version 3.8.1 (5). A total of 2,949,300 reads were assembled into 20 contigs with an *N*_50_ length of 461,990 bp and a largest length of 783,435 bp. This assembly resulted in a draft genome sequence of 2,169,598 bp with a G+C content of 41.3%. A total of 2,099 protein-coding DNA sequences and 46 tRNAs were detected by Prokka (6) and the RAST server (7).

Previous studies of termite gut isolates of lactococci have suggested that they play a role in the fermentation of degradation products of (hemi)cellulose, in the oxygen reduction accompanying the shift from lactate to acetate during fermentation (8), and in the supply of folate, a cofactor essential for acetogenesis from CO_2_ and H_2_ and required by acetogenic treponemes in the gut (9). For the last role, the genome annotation indicated the presence of genes for folate biosynthesis: *folE*, *folB*, *folK*, *folP*, *folC*, and *folA*; the *folQ* gene encoding dihydroneopterin triphosphate diphosphatase was not indicated, although a diphosphohydrolase-like gene was fused in frame with the *folE* gene. The genome sequence will facilitate further studies on the symbiotic roles of *Lactococcus* sp. Rs-Y01 in the termite gut.

### Accession number(s).

The genome sequence of *Lactococcus* sp. strain Rs-Y01 has been deposited at DDBJ/GenBank under the accession numbers BEDT01000001 to BEDT01000020.
